# Enhancer Runaway and the Evolution of Diploid Gene Expression

**DOI:** 10.1371/journal.pgen.1005665

**Published:** 2015-11-12

**Authors:** Frédéric Fyon, Aurélie Cailleau, Thomas Lenormand

**Affiliations:** UMR 5175 CEFE, CNRS, Université Montpellier, Université P. Valéry, EPHE, Montpellier, France; University of Michigan, UNITED STATES

## Abstract

Evidence is mounting that the evolution of gene expression plays a major role in adaptation and speciation. Understanding the evolution of gene regulatory regions is indeed an essential step in linking genotypes and phenotypes and in understanding the molecular mechanisms underlying evolutionary change. The common view is that expression traits (protein folding, expression timing, tissue localization and concentration) are under natural selection at the individual level. Here, we use a theoretical approach to show that, in addition, in diploid organisms, enhancer strength (i.e., the ability of enhancers to activate transcription) may increase in a runaway process due to competition for expression between homologous enhancer alleles. These alleles may be viewed as self-promoting genetic elements, as they spread without conferring a benefit at the individual level. They gain a selective advantage by getting associated to better genetic backgrounds: deleterious mutations are more efficiently purged when linked to stronger enhancers. This process, which has been entirely overlooked so far, may help understand the observed overrepresentation of cis-acting regulatory changes in between-species phenotypic differences, and sheds a new light on investigating the contribution of gene expression evolution to adaptation.

## Author Summary

With the advent of new sequencing technologies, the evolution of gene expression regulation is becoming a subject of intensive research. In this paper, we report an entirely new phenomenon acting on the evolution of gene regulatory sequences. We show that in a small genomic region around genes there is a selection pressure to increase expression, such that stronger enhancers are favored. This leads to an open-ended escalation of enhancer strength. This outcome is not a particular case and we expect it to occur for all genes in nearly all eukaryotic diploid organisms. We also show that this escalation is not stopped by stabilizing selection on expression profiles. Indeed, regulators may coevolve to maintain optimal phenotypes despite the enhancer strength escalation. This widespread phenomenon can significantly shift our understanding of gene regulatory regions and opens a wide array of possible tests.

## Introduction

The evolution of gene expression has become a subject of intensive research in the last years, sparking debates upon its role in adaptive evolution and speciation [1–6]. Clearly, protein folding, expression levels, timing and tissue localization of expression are important regulatory traits under selection as they are essential steps in the genotype-to-phenotype map.

Gene expression is regulated at each step along the pathway from DNA to protein. Among them, transcription initiation is a crucial step responsible for a large proportion of the variation in expression profiles. Here we will focus on regulatory regions controlling this transcription initiation. Changes in gene expression are often caused by mutations in *cis*-regulatory elements (CREs) and trans-regulatory elements (TREs) [7]. *Cis* and *trans*-regulators control transcription of genes located on the same chromosome, or on both homologous chromosomes, respectively. Several recent technological breakthroughs [7–10] have considerably improved our ability to study these regulatory sequences and their associated expression profiles. They revealed that gene expression profiles were highly variable and heritable within species [2,11,12], quickly diverging among species [8]. Furthermore, many studies have shown how changes in gene expression contribute to adaptive changes, by natural selection at the individual level [4,6,13–17]. From a theoretical standpoint, several models have also been developed to understand the evolution of gene expression by individual level selection [18–22].

In this paper we investigate a new and different selective phenomenon also acting on the evolution of regions controlling transcription initiation. This phenomenon may contribute to the fast divergence of regulatory networks between closely-related species, but unlike the usual view, it is not rooted in individual-level selection, and hence does not necessarily increase individual fitness. We term this selective process ‘ER‘ (for ‘enhancer runaway’). It results from competition for expression between homologous cis-acting regulatory sequences in diploid organisms. With this gene-level selection, these regulatory sequences behave as self-promoting genetic elements.

Transcription initiation is determined by the binding of a suitable RNA-polymerase to a Transcription Start Site (TSS). This binding involves a complex machinery of often over 30 partner proteins [23]. It depends on the interaction of CREs and TREs. CREs are non-coding sequences located on the same chromosome as the regulated gene. They include core promoters located around the TSS, which integrates the regulatory inputs [24]. They also include enhancers, which influence transcription initiation rate independently from their orientation or localization on this chromosome [9]. TREs are coding sequences, located anywhere in the genome, that produce transcription factors (TFs), which bind to CREs on both homologs. They can also produce cofactors, which bind other proteins (including other TREs) [7].

In order to show how the ER process works, we use population genetic models with both protein-coding sequences (the ‘gene(s)’) and regulatory sequences (enhancers or transcription factors). For generality, we do not specify precisely how regulatory mutations change regulatory networks. We simply assume that mutations occur in enhancers and TFs, which impact expression levels. Indeed, there are many ways for an enhancer mutation to modify expression levels [2,7,9,25,26]. It may change for instance the binding site affinity towards TFs, the number and/or spacing between binding sites, or the nucleosome conformation around the enhancer region, which strongly impacts DNA accessibility for the transcriptional machinery. Because enhancers act in *cis*, the presence of different alleles at the enhancer locus creates a pattern of allele-specific expression (imbalance in chromosomal origin of transcripts) for the protein-coding gene [27,28]. Indeed, in heterozygous individuals, the ‘weaker’ enhancer contributes less to protein expression than its ‘stronger’ counterpart on the other chromosome, causing imbalanced expression of homologous gene alleles. TF mutations may also alter expression levels in various ways. For instance, they can change its DNA-binding domain (modifying its affinity for different binding sites) or its protein-protein binding domain (modifying its interactions with e.g. cofactors or histones). However, they act in *trans*, and do not generate allele-specific expression.

To obtain a good understanding and broad evaluation of the significance of the ER process, we investigate several complementary models. In a first model, we study the ER process in the absence of individual level selection for expression level. We then incorporate individual level selection on expression. Many form of individual level selection on expression levels could have been chosen, and there has been indeed much debate over the different selection pressures acting on expression levels. While some argue that regulatory polymorphism is mainly neutral or quasi-neutral [29,30], others suggest that regulatory evolution is mostly shaped by stabilizing selection [31–33] and occasionally by directional selection [8,34–36]. In a second and third model, we thus chose to introduce stabilizing selection on expression levels (due to a relative gene dosage constraint in model 2, or an absolute expression constraint in model 3). In these two models, other regulatory regions can evolve in concert (other enhancers in model 2, TFs in model 3). Finally, since the ER process may act very differently in genomes exhibiting inbreeding and low heterozygosity, we investigate how it is influenced by the mode of reproduction (inbreeding in model 4). Overall we show that, in a small genomic region around genes, there is a selection pressure on enhancer to increase expression levels. This phenomenon leads to an open-ended escalation in enhancer strength (a ‘runaway’). This process is not halted by inbreeding, or by stabilizing selection on expression levels, as long as enhancers can evolve in concert with other regulatory sequences (enhancers or TFs) involved in the same regulatory network. Enhancer runaway is not a highly specific or idiosyncratic process: it is expected to occur at variable intensities for all genes in nearly all eukaryotic diploid organisms. This widespread phenomenon may significantly shift our current understanding of gene regulatory regions, and opens a wide array of possible tests and comparisons.

## Model

### Competition for expression

To illustrate how competition for expression works, we first present a two-locus model in an infinite, diploid, sexual population. The first locus, the ‘gene’, codes for a protein. We suppose that it undergoes recurrent deleterious mutations (with fitness effect *s* and dominance *h*) at a rate *u*, but the argument would apply equally well with beneficial mutations (see methods). This locus quickly reaches the usual deterministic mutation–selection equilibrium, with deleterious mutation at frequency *u*/*hs*. The second locus, the ‘enhancer’, is located at a recombination distance *r* and controls the expression of the gene in a cis-regulatory fashion. We wish to determine the selection pressure acting on mutations that modify the strength of this enhancer.

In model 1, we consider that the overall expression level is tightly controlled, for instance because of trans-acting regulatory factors producing a negative feedback loop. Such a feedback loop is not relevant to all genes, but is a particularly useful starting case. It allows investigating how the ER process works without the complication of additional selection pressures acting, at the individual level, on protein expression. We thus assume that overall expression levels are constant (due to the feedback loop), such that only the relative contributions of each homologous alleles vary due to mutations on enhancers.

Labelling *e*_1_ and *e*_2_ the strengths of the two enhancer alleles of an individual, the gene associated with enhancer of strength *e*_*1*_ contributes a fraction *e*_1_/(*e*_1_+*e*_2_) of proteins produced. As a consequence, the gene allele linked to a stronger enhancer contributes a larger share of proteins.

**Fig 1:**
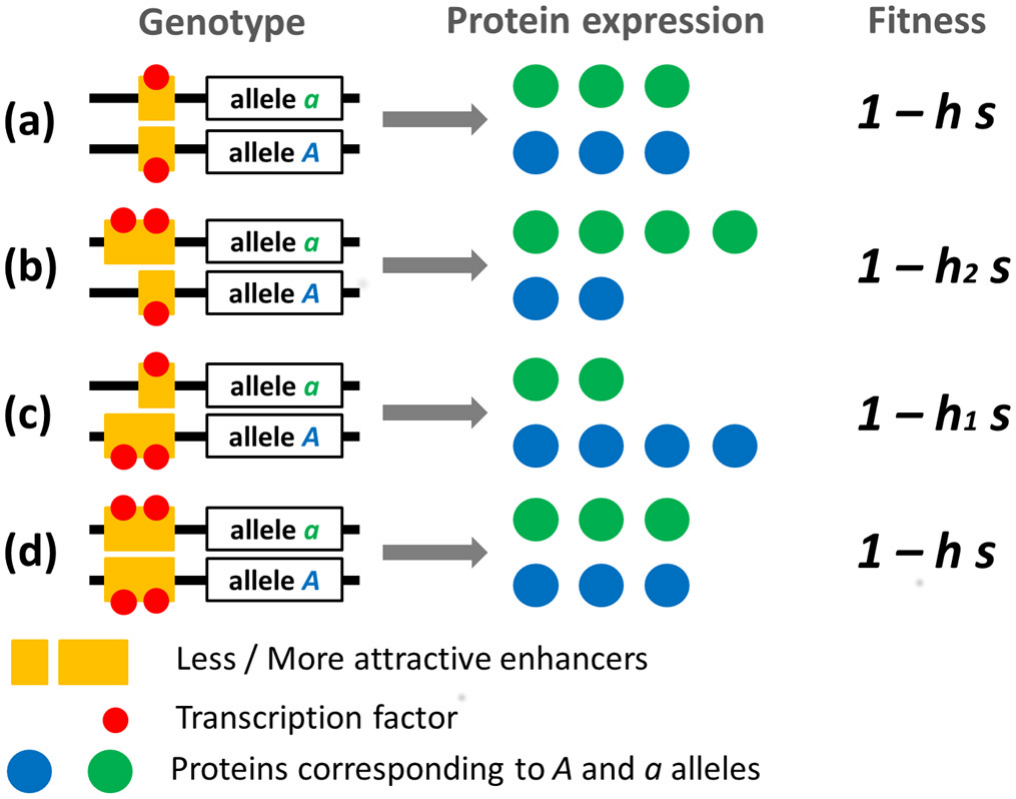
Schematic representation of protein expression and fitness of gene heterozygotes. Here, the strengths of two enhancer alleles are represented by their ability to attract transcription factors. Four genotypes are represented: weaker enhancer homozygote (a), stronger enhancer homozygote (d) and enhancer locus heterozygotes (b) and (c). In enhancer locus heterozygotes, the stronger enhancer is either associated with the deleterious gene allele (b) or with the viable gene allele (c). Corresponding fitnesses are indicated. Note that we consider here a case where the total amount of proteins produced is constant.

In double heterozygotes, if the deleterious allele of the gene is linked to the weaker enhancer, less than 50% of the proteins produced will be of the defective form, and thus its effect on fitness will be less than predicted based on its dominance coefficient *h* (which in effect reduces its dominance to *h*_1_<*h*). In contrast, if it is linked to the stronger enhancer, its deleterious effect on fitness will be stronger than predicted by *h* (which in effect increases its dominance to *h*_2_>*h*). The fitness effect of linkage with a specific enhancer can thus be modeled as a change in *h* that occurs only when the enhancer is heterozygote, while dominance is necessarily *h* as long as an enhancer allele is fixed, as illustrated in Fig 1.

**Fig 2:**
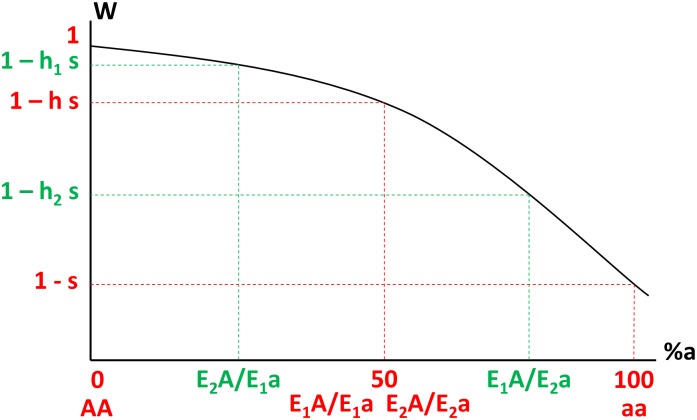
Variation of fitness (W, y-axis) as a function of the percentage of defective proteins noted %a (x-axis, from 0% in AA homozygotes to 100% in aa homozygotes) in different genotypes, where E1 and E2 are the weaker and stronger enhancer alleles respectively (e_2_ > e_1_). This function is necessarily monotonic and concave when deleterious mutations are recessive (h < ½).

Because deleterious mutations are usually partially recessive [37], the relationship between the fraction of defective proteins and fitness is necessarily nonlinear, monotonously decreasing and concave (Fig 2). Thus, we have *h*_1_ + *h*_2_ > 2*h* (applying Jensen’s Inequality to the strictly concave fitness function). In other words, a polymorphism at the enhancer locus necessarily increases average dominance (the mean of *h*_1_ and *h*_2_ is greater than *h*), which increases the strength of selection against heterozygous deleterious mutations, but also reduces the fitness of individuals carrying these mutations.

The deterministic change in frequency at the enhancer locus can be computed from standard population genetics equations, considering a generic diploid life cycle with four steps: diploid selection, meiosis with recombination, mutation and syngamy. This frequency change can be decomposed into two terms that correspond to ‘masking’ and ‘purging’, as introduced in previous related models [38,39] (see derivation in methods). The ‘masking’ term does not depend on recombination, and is frequency-dependent. It always disfavors the enhancer allele when rare, independently of its strength. The reason is that a rare enhancer will most often be represented in double heterozygotes (since deleterious mutations often are rare too), which have lower average fitness, since they have higher average dominance (this is similar to Fisher’s argument for the evolution of lower dominance [40]). In other words, rare enhancer alleles pay the cost of unmasking deleterious alleles. This term is strongly conservative, as it prevents any new enhancer to enter the population. The ‘purging’ term, in contrast, is frequency-independent, always favors stronger enhancers, and increases when recombination between the enhancer locus and the gene decreases. The reason is that genes that are linked with stronger enhancers are more exposed to selection and more efficiently purged from deleterious mutations. Hence, stronger enhancers are disproportionately found on –and hitchhike with– favorable genetic backgrounds. Overall, combining these two effects, weaker mutant enhancers are always disfavored, while stronger mutant enhancers are favored if sufficiently tightly linked to the gene for the ‘purging’ effect to overcome the ‘masking’ effect. The recombination distance where the two effects balance each other depends however on the strength difference of the two enhancer alleles. For enhancers that are very similar in strength, the range of recombination distances where the runaway can occur is larger, but the intensity of selection on the stronger enhancer is weaker (S1 Fig). This is due to the fact that, for small differences in enhancer strength, *Δh* becomes vanishingly small compared to *δh* (and thus the masking term in front of the purging term, see Eqs 3 and 5 in methods). Overall, stronger runaway is expected close to genes because selection intensity on new enhancers is stronger at small recombination distances and because the runaway only concerns enhancers of small effects at larger recombination distance.

**Fig 3:**
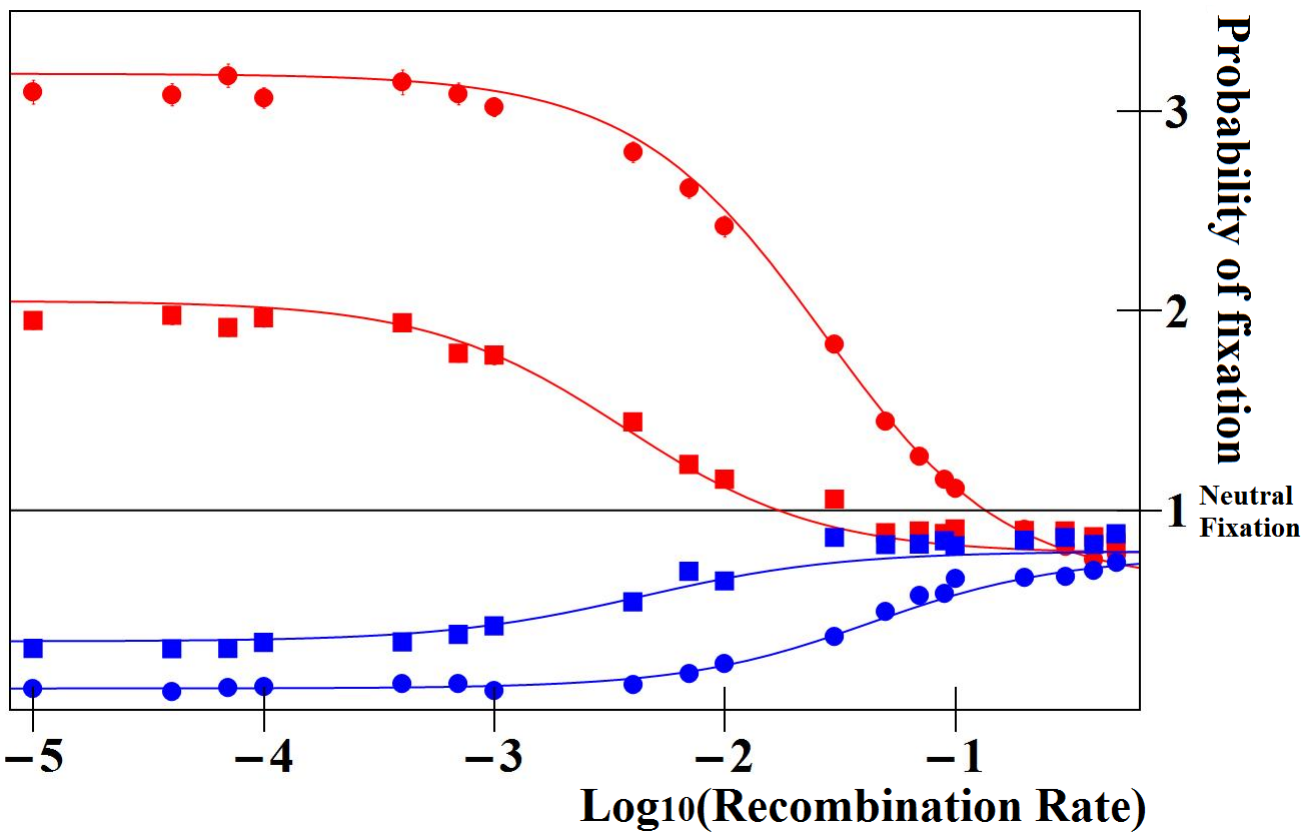
Ratio of fixation probabilities of mutations altering enhancer strength relative to that of a neutral mutation. In red, the mutant enhancer is three times stronger than the wild type; and in blue, three times weaker. Simulated and analytically predicted values (see methods) are represented by dots and lines, respectively. Values are reported for the case where enhancer strength evolution does not alter overall protein expression (model 1, see text), for various recombination rates between the enhancer and the gene (x-axis), for weak (*s* = 0.01, squares) or strong selection (*s* = 0.1, circles) and partial recessivity (*h* = 0.25). Results are illustrated for *N*_pop_ *u* = 1 (where *N*_pop_ is the population size and *u* the gene mutation rate). Results for higher *N*_pop_ *u* just need to be multiplied by a factor equal to *N*_pop_ *u*. For instance the relative fixation probability for *N*_pop_ *u* = 10 of tightly linked stronger enhancers would be ~20 or ~30, for *s* = 0.01 or *s* = 0.1, respectively. Weaker enhancers (blue) are always selected against, while stronger enhancers (red) are selectively favored provided that they are closely linked to the gene, and disfavored otherwise.

In regulatory regions close to the gene, selection thus favors enhancers contributing more to protein production: homolog enhancers compete for expression. This outcome is illustrated on Fig 3, where this analysis is checked against –and agrees with– stochastic numerical simulations reporting the fixation probabilities of new enhancers in finite populations.

This finding indicates that stronger and stronger cis-regulatory elements should evolve in an open-ended fashion in the vicinity of genes, as long as that does not influence the total amount of proteins produced. Competition for expression may thus be responsible for a runaway process of enhancer strength. During this process, allele-specific expression would transiently occur, but expression balance would be restored once the new enhancer reaches fixation. This process shares many similarities with the endless occurrence and spread of new segregation distorters that transiently bias Mendelian ratio while they sweep [41]. It also shares similarities with models of Fisher runaway in the context of sexual selection, where female mating preference drives the open-ended evolution of extravagant traits in males [42].

Interestingly, the spread of such stronger enhancers occurs even though it temporarily decreases population mean fitness (see methods). Indeed, stronger enhancers spread because they find themselves on better backgrounds, but at the expense of temporarily increasing mean dominance and hence unmasking deleterious mutations. Overall the ER process does not optimize mean individual fitness in the population.

### Competition for expression and stabilizing selection on expression levels

Gene expression regulation is not necessarily embedded in a negative regulatory loop, as considered above. Mutations on enhancers will often alter overall expression levels. When this occurs, stabilizing selection on overall gene expression will interfere with competition for expression. We developed two additional models (model 2 and 3) with such interactions (see methods). In these models, mutations on enhancers alter both relative contribution to expression, *e*_1_/(*e*_1_+*e*_2_), and total expression levels, *e*_1_+*e*_2_. We assume that total expression levels undergo stabilizing selection with different intensities that reflect the functional diversity of genes (over- or under-expression may be more costly for some genes than others).

**Fig 4:**
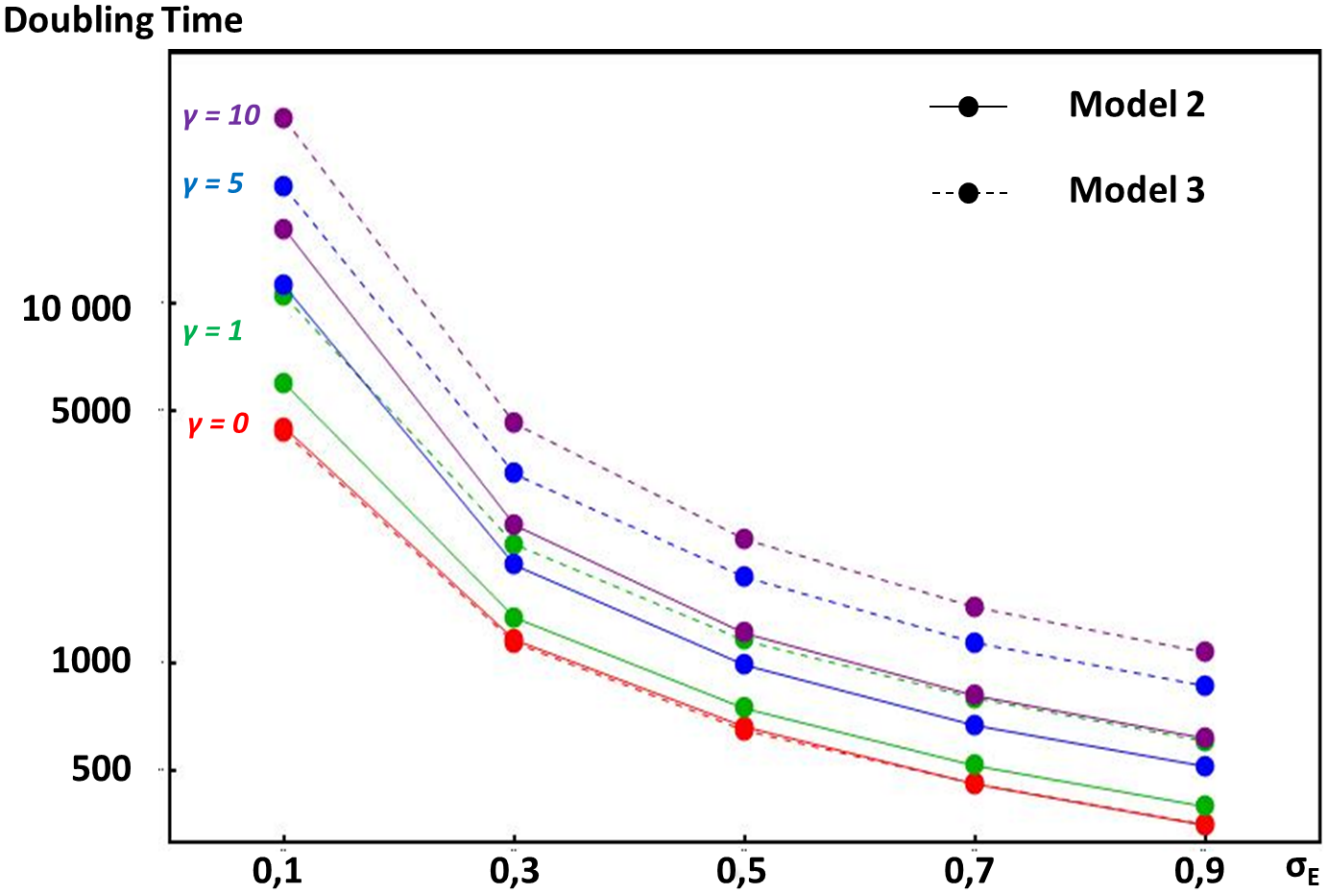
Comparative doubling times of enhancer strength escalation in models considering different selection pressures acting on overall gene expression. Y-axis indicates doubling times of enhancer strength (the expected number of generations needed to double the initial enhancer strength) according to the mutation size standard deviation on enhancers (x-axis). Because in all models presented, enhancer strength increases open-endedly in average, this doubling time measures the rate of escalation (see methods). The mutation size standard deviation on enhancer strength (*σ*_*E*_) is a measure of the magnitude of mutational input (see methods). In model 2 (plain lines), overall expression of one gene is involved in a dosage relationship with the overall expression of another gene. Overall expression is determined by one enhancer locus per gene, and any departure from optimal dosage is costly. In model 3 (dashed lines), the absolute amount of protein produced is under stabilizing selection, but expression level is influenced by both an enhancer and a TF locus. In both models, stabilizing selection intensity is scaled by the intensity of selection at the gene locus (see methods). Deceleration of ER process due to stabilizing selection is illustrated for *γ* values equal to 0 (in red), 1 (in green), 5 (in blue) and 10 (in purple). Doubling times were obtained using stochastic simulations (see methods).

In model 2, we assume that stabilizing selection on expression levels stems from gene dosage, such that the optimal amount of a given protein depends on the amounts of another protein coming from another loci, as occurs for instance in enzymatic or metabolic pathways. This produces the strongest constraint on enhancer strength evolution when only two proteins are concerned (see methods). In this case, any increase in the expression of gene 1 causes a departure from optimal dosage for gene 2, which reduces fitness. Results show that such stabilizing selection fails to prevent enhancer strengths from escalating. This is because enhancers of both genes coevolve: their strengths increase in parallel, allowing maintenance of the correct protein dosage. However, stabilizing selection tends to decrease escalation rates (longer doubling times on Fig 4, see methods). Strength-increasing mutations of large effects are indeed counter-selected, since they lead to large deleterious departures from optimal gene dosage. However, strength-increasing mutations of small effects lead only to small enough departures from optimal gene dosage for the genotype to survive until optimal gene dosage is restored by compensatory mutations. Stabilizing selection needs to be relatively strong (stronger than the selection at the gene locus) for it to significantly alter escalation rates.

In model 3, we considered a situation where stabilizing selection acts directly on the absolute expression levels of a gene, but transcription factors influence expression levels as well as enhancers. We designed a three-locus model with a gene, an enhancer locus, and a TF locus, where both the strength of the enhancer and TF combine to determine expression levels (see methods). Here again, as shown on Fig 4, stabilizing selection slows down but does not stop the ER process, due to coevolution between regulators (here, enhancers and TFs). As enhancer strength increases, TF strength decreases in proportion, which maintains approximately constant and optimal total expression levels. In this model, escalation rates are systematically lower than the ones obtained with model 2 (except for *I = 0*). This is to be expected as two identical enhancer loci are exposed to the ER process in model 2, while only one enhancer is exposed to the ER process in model 3 (the TF locus only responds to stabilizing selection). As a consequence, in model 3, stabilizing selection importantly decrease escalation rates at lower intensities (lower than the intensity of selection at the gene locus).

### Impact of mating system on competition for expression

Variation in enhancer strength only makes a phenotypic difference in double heterozygotes. In situations where such heterozygotes are less frequent than under random mating, the pace of the ER process is expected to be slower. Inbreeding is a typical and common situation decreasing heterozygote frequency. It may be caused by various processes (e.g. selfing in hermaphroditic plants or animals, population structure). To determine whether ER indeed differs among species exhibiting e.g. different mating systems, and to quantify the extent of this reduction, we introduced partial selfing in the first model.

**Fig 5:**
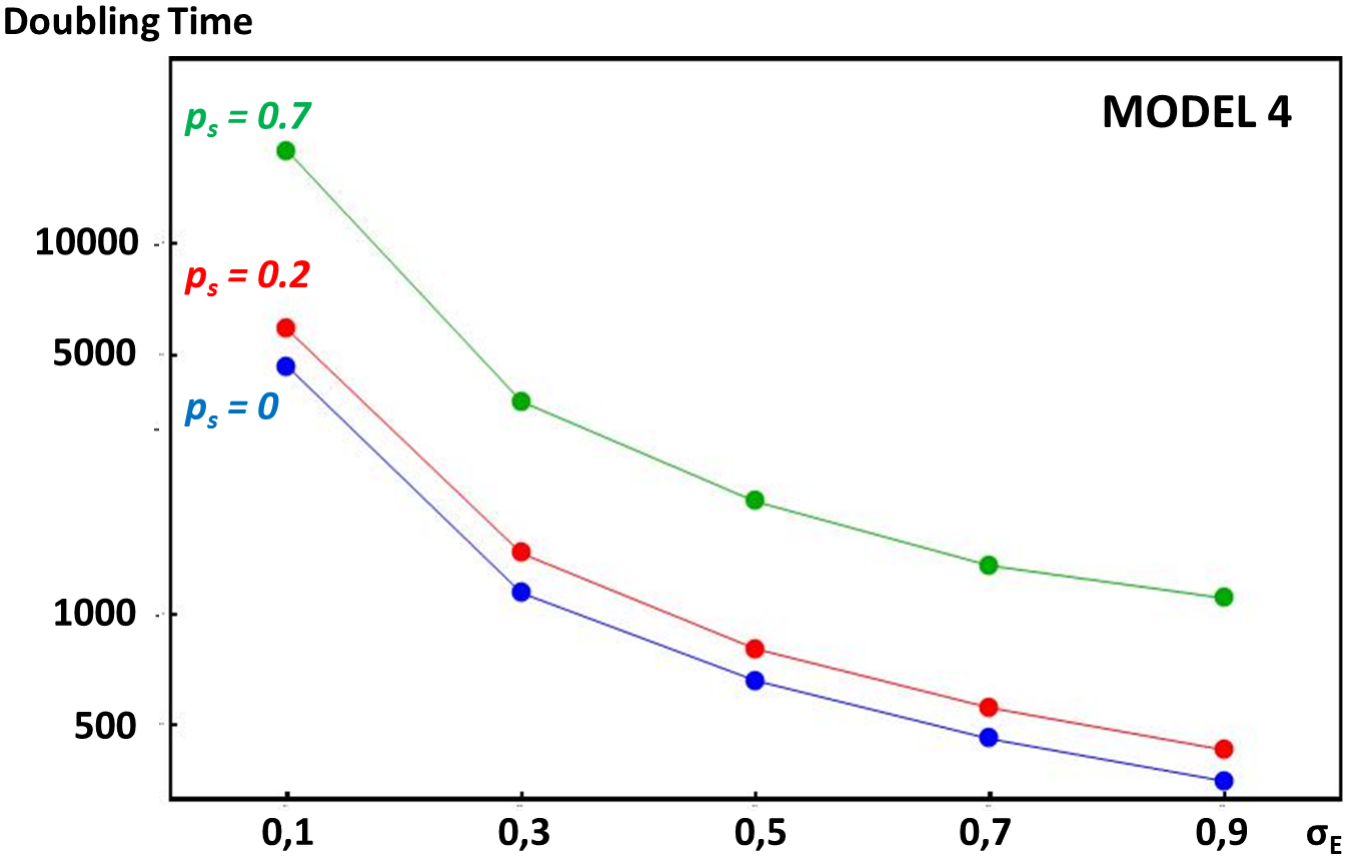
Comparative rate of enhancer strength escalation in models with or without self-fertilization. Axes and simulation methods are the same than for Fig 4. Selfing rate *p*_*s*_ is 0, 0.2 and 0.7 on blue, red and green curves, respectively. In all three cases, enhancer strength escalation is faster with larger mutational variance σ_E_^2^ on the enhancer locus. Self-fertilization slows down the ER process.

In model 4, at each generation, individuals were considered to have probability *p*_*s*_ of selfing (i.e. with probability *p*_*s*_, the same parent is used to sample the second gamete). As expected the ER process slowed down as selfing rate increased. Fig 5 illustrates this behavior. Results show that relatively high levels of self-fertilization are needed for the ER process to be significantly slowed down.

## Discussion

The ER process we describe sheds a new light on the evolution of gene regulatory regions in diploids. It should be a widespread phenomenon, applicable to all genes in diploid eukaryotes as the theory only involves generic assumptions (recessivity of deleterious mutations [37], genetic variation at enhancer loci [43], CREs—TREs coevolution [44], stabilizing selection on expression levels [31–33]). This process has been acting probably since early unicellular diploids, more than a billion years ago, before the evolution of complex combinatorial or developmental regulatory pathways. As we already stressed in the introduction, it does not undermine the idea that expression regulation is also subject in contemporary eukaryotes to other important selective effects (direct selection for expression level, timing and localization).

Before discussing the implications and predictions associated with this theory, it is useful to relate it to previous models involving variation in dominance, ploidy, mutation load and selection for modifiers. It shares with models of ploidy evolution the fact that increased haploid expression leads to more efficient purging of deleterious mutations, which benefits tightly linked modifiers [38,39]. It shares with models of dominance evolution [45–48] the limit that selection on modifiers is weak, of the order of the mutation rate (even if this issue may be alleviated if migration is the source of deleterious alleles [48,49]). Such a weak selection is usually seen as a limit in the case of dominance evolution because genetic drift in small populations or small pleiotropic effects of modifiers should easily overwhelm the selection pressure for dominance modification [50]. A distinctive feature of our theory is that the ER process is not strongly limited by such pleiotropic effects: as we showed, direct selection against suboptimal expression levels can be readily compensated by CREs-CREs or CREs-TREs coevolution, without halting the ER process. A critical difference with previous models is that dominance does not evolve: the dominance of deleterious mutations is *h* before the sweep of a stronger enhancer, and is still *h* after its fixation. Similarly, ploidy or the number of gene copies stays constant and does not evolve (with the consequence that, unlike in models involving gene duplicates or ploidy variation, the mutational load is not permanently changed by a change in gene number [38,47]). In our models, selection is frequency-dependent at leading order and leads to a runaway escalation not seen in models of ploidy or dominance evolution. Our models specifically focus on cis- or trans-acting modifiers, mimicking the actual genetic variation occurring on regulatory regions. Cis-regulation introduces naturally an asymmetry in the fitness of the two double heterozygotes (EA/ea and Ea/eA), which is also not a feature present in models of ploidy or dominance evolution.

This theory leads to a series of predictions that can be further tested. We highlight eight of them below using capital letters A-H. The ER process should occur for almost all genes in diploids, but with different, and possibly very different rates depending on their specific evolutionary constraints (regulatory loops, dosage relationships, intensity of stabilizing selection). For instance, as we illustrated with our first model, this runaway is fastest for enhancers that are embedded in a downstream negative regulatory loop (prediction A). Such negative feedback loops have been extensively described and are often thought to largely contribute to phenotypic robustness (e.g. [51–53]). This runaway should be slower for genes that are not regulated with such loops and exposed to direct stabilizing selection on relative or absolute expression levels. The pace of the ER process depends in those cases on the form and intensity of the stabilizing selection, as well as on the opportunities for CREs and TREs to coevolve to maintain optimal total expression levels, which may differ among genes.

Our results show a strong impact of the recombination rate between the enhancer and the gene on the ER process (see Fig 3). There are two regimes: (1) for large recombination rates, the ER process does not occur (large and medium effect enhancers are selected against and small effect enhancers are nearly neutral), while (2) at shorter genetic distances, the runaway occurs and its rate increases as the recombination rate decreases. Everything else being equal, the ER process should cause a positive correlation between enhancers’ strength and their proximity to the gene, but it should not concern enhancers located at large genetic distances (prediction B). This prediction would be consistent with the observation that most CREs remain close to the gene they regulate [54]. The critical genetic distance delimiting the two ER regimes increases as the intensity of selection on the gene increases. As a result, genes undergoing stronger purifying selection would be expected to have a larger surrounding region where enhancers increasing expression may arise and compete for expression. Consequently, we predict that genes experiencing stronger purifying selection should exhibit a larger surrounding regulatory region, and that, for a given genetic distance to such genes, enhancers should exhibit a faster ER process (prediction C). Such qualitative predictions may be altered if there is an inherent physical tendency for CREs strength to covary with physical map distance. However, if, as is most likely, CRE strength decreases with physical distance along the chromosome, the qualitative pattern will remain identical (since decreased CRE strength and increased recombination both prevent the fixation of new enhancers).

There are several reasons, not included in our models, why the ER process could slow down or stop. First, many mutations on enhancers are likely to have pleiotropic effects on timing or localization of expression. These features are not included in our model, and probably limit the availability of enhancer mutations that can contribute to the ER process in higher eukaryotes. Second, mutations may not be able to increase enhancer strength indefinitely. For instance, when a binding motif sequence is optimized for a particular TF, there may be little room for further improvement. Similarly, there is a limit to the number of binding motifs that can be packed in a regulatory region, etc. These constraints could be revealed by studying the effect of random mutations on regulatory regions. If enhancer strength is maximized by the ER process, no (or very few) random mutations on the enhancer will be able to increase its strength, such that the strength of those enhancers should be biased downward by random mutations. Conversely, if a TF has evolved to compensate for the enhancer runaway (i.e. by increasingly repressing transcription), random mutations on this TF should on average increase expression levels (prediction D).

The evolution of regulatory networks’ complexity is not well understood and controversial (some argue that the evolution of complex regulatory networks stem from adaptive processes [18,20,55], while others consider a non-adaptive origin [56,57]). We saw that the ER process depends on the shape of regulatory networks (e.g. presence of feedback loops, of gene dosage etc.). It may also be facilitated with more complex regulatory architecture, as highly degenerate and complex regulatory architectures [58] could provide more ‘degrees of freedom’ for CREs—TREs (co)evolution. Interestingly, the ER process may also contribute to the evolution of complex regulatory networks. For instance, an increase of enhancer strength may be achieved e.g. by locally duplicating TF binding motifs (and hence increasing the complexity of regulatory architecture). The duplicated motifs may further diverge to attract a larger diversity of TFs if this happens to be a route to further increase of enhancers’ strength: combinatorial regulation (where expression specifity results from a particular combination of several TFs, as in e. g. [59]) may thus evolve from the ER process. A positive feedback loop may thus occur between the ER process and the evolution of regulatory complexity. Much of these effects will rely on the contingency of mutational variation on enhancers, but can produce a level of architecture complexity much beyond the level expected under individual selection alone. Species where the ER process is expected to be faster (e.g. outcrossing vs. selfing diploids, diploid versus haploid eukaryotes) should exhibit more complex regulatory architectures (prediction E). Like for other theories for the evolution of complexity [56,57], complexity would emerge here as a by-product of another process (here ER) and is not a direct target of selection.

It is generally envisioned that many regulatory networks can be functionally equivalent. For instance, many CREs—TREs combinations can perform the same signaling, and different global regulatory wirings among TREs and CREs can achieve the same regulatory pattern. Like with a key / lock or signal / receiver mechanisms, the central functional requirement is the reciprocal recognition of interacting regulatory elements. Such a situation produces a fitness landscape with a ridge, along which ‘evolutionary freedom’ allows for substantial neutral divergence [60]. This process is often thought to drive relatively fast divergence of regulatory networks among species without modifying expression patterns much [61]. In our models, the same process occurs (coevolution between regulators occurs without modifying much expression levels), but, in addition, selection for stronger proximal CREs leads to faster-than neutral divergence (prediction F). Using again the fitness landscape metaphor, it means that the ridge is actually not flat, but is slightly sloping: evolution between networks with the same expression pattern is not neutral, but rather directed to favor networks involving stronger proximal enhancers. Moreover, this faster-than-neutral divergence is expected to be accentuated for CREs (compared to TREs, prediction G). Indeed, the ER process necessarily involves CREs, and optionally TREs. For instance, cis- cis- coevolution illustrated in model 2 does not involve TREs evolution. This may partly explain why CREs are usually (but not always, e.g. [62]) found to contribute more than TREs to expression regulation divergence among species (e.g. in *Saccharomyces* [63], *Drosophila* [64], *Gasterosteus* [16] and *Mus* [65]).

As shown in model 4, the ER process is slower in presence of inbreeding. In particular, the ER process is expected to be faster in outcrossing than in self-fertilizing species (prediction H). One way to test for this prediction would be to compare CRE strengths in hybrids between closely related outcrossing and self-fertilizing species. Indeed, in the hybrid, expression level differences between alleles can only be explained by CRE variation, as TREs from both parents are shared [66]. Such method could be used in e.g. Arabidopsis [67] or Capsella [68]. The expectation would be that CREs derived from the self-fertilizing species should be weaker on average, biasing expression pattern in F1s towards the outcrossing parent alleles. Such test would require however to control for the direction of the cross (distinguishing the species effect from maternal versus paternal effects).

Several genetic oddities that have been observed in some species may greatly limit the ER process. For example, in somatic cells of Diptera, homolog chromosomes pair and expression is largely influenced by a phenomenon referred to as ‘transvection’ [69]. With transvection, “CREs” impact regulation of both homolog chromosomes (i.e. are not really behaving as cis-regulatory elements as they also regulate in *trans*). Somatic pairing and transvection in these taxa certainly reduce, or even eliminate competition for expression. Similarly, repulsion of homologs, as found in mammals’ nucleus [70], (i.e. the fact that the chromosome territories of homologs tend to be further apart within the nucleus compared to a random pair of chromosomes) could also strongly reduce competition for transcription factors between competing CREs, as the transcription of homologs likely involves different and spatially segregated ‘transcription factories’ [71]. In both cases, reduced competition for expression between homologs could strongly limit the ER process. Finally some recombination hotspots are located close to genes in several species [72,73]. This could also strongly limit the ER process by breaking linkage disequilibria between CREs and genes. Whether some of these genetic oddities evolved as suppressors of the ER process is an intriguing possibility, especially given that their evolutionary significance remains elusive.

## Methods

We describe below the models (1–4) we designed to infer enhancer strength evolution. These models differ by the mode of reproduction and the type of stabilizing selection acting throughout gene regulatory networks. In model 1, we study the evolution of enhancers assuming that expression levels are so tightly controlled that the total amount of proteins is strictly constant. There is no selective pressure resulting from changes in overall protein expression. In such a situation, a competition for expression appears between homolog enhancers. We used this model to understand the genetic cause of the resulting ER process and the evolutionary patterns it creates. While this model is relevant for genes with regulatory loops (and for understanding selection pressure acting on expression balance), it may not capture all selection pressures acting on enhancers. Thus, in following models (2 and 3), we consider that enhancers do influence the total amount of protein expressed and investigate how it interacts with the selection pressure described in model 1. We consider two cases. In model 2, there is an optimal dosage between expression levels of different genes: enhancer strength variation on one gene causes a departure on gene expression dosage, which is deleterious. In model 3, the fitness penalty arises from any departure from an absolute optimal expression level, but expression levels depend on enhancers and transcription factors, and not only on enhancers as in previous models. Finally in model 4, we introduce partial selfing in a model 1 genetic setup, to infer the effects of inbreeding on the ER process.

### Designing model 1

We firstly consider a diploid two-locus model: the first locus, referred to as ‘the gene’ is a protein-coding locus undergoing mutations and diploid viability selection; the second one, referred to as ‘the enhancer’, is a CRE locus controlling the expression of the gene. The gene is exposed to recurrent deleterious mutations, at a rate *u* per individual per generation, changing *A* alleles into *a* alleles. In the analytical derivation below, we focus on this bi-allelic case, but this assumption is relaxed later for numerical simulations. We define the relative fitness of genotypes as *1*, *1 –h s* and *1 –s* for *AA*, *Aa* and *aa* genotypes, respectively, where *s* is the selection coefficient against the *a* allele and *h* its dominance. The enhancer is at a recombination distance *r* from the gene. We consider two alleles (*E*_1_ / *E*_2_), which differ by their ability to promote expression of the gene located in *cis* (i.e. on the same chromosome). This ability is referred to as their ‘strength’ and noted *e*_1_ and *e*_2_. Biologically this strength depends on many parameters, such as sequence affinity, chromatin state, binding network or intracellular signals [7]. Furthermore, different mechanistic models have been put forward to describe enhancer effects: they are thought to change either promoter activation levels or the probability that a promoter will be activated to initiate transcription [74]. Here, we will not assume a particular mechanism but cover all these possibilities by simply supposing that enhancer sequences intrinsically differ by their ability to activate gene transcription.

We assume that the gene on the same chromosome than an enhancer with strength *e*_1_, facing on the homolog chromosome an enhancer with strength *e*_2_, contributes to a fraction *e*_1_/(*e*_1_ + *e*_2_) of protein produced (see Fig 1). As a consequence, the gene associated with a stronger enhancer contributes a larger share of proteins. A major feature of this first model is that the total protein expression level is tightly regulated: the total amount of protein produced is assumed to be constant, and does not depend on enhancers’ strength (6 proteins are always produced on Fig 1). Such a situation would occur, for instance, when the amount of transcription factors, or a repressor is regulated through a negative feedback loop (e.g. through the total amount of protein produced or by the concentration of a downstream metabolite resulting from protein activity). Besides representing this fairly common biological situation, this model is also important to understand the evolution of enhancer strength independently from the selection pressure acting on the amount of protein in a given cell at a given time.

When the gene locus is homozygous, the fitness of individuals does not depend on the enhancer alleles, as 100% of the proteins are of the same type (the fitness is *1* or *1 –s* for *AA* and *aa* genotypes respectively). However, different situations arise in individuals that are heterozygous at the gene locus. When they are homozygous at the enhancer locus, they equally express each type of proteins (50% each), and their fitness is *1 –h s* (genotypes (a) and (d) in Fig 1). When they are heterozygous at the enhancer locus they express more (or less) of the defective protein if the stronger (or weaker) enhancer is associated with the deleterious allele (genotypes (b) and (c) in Fig 1). This changes dominance at the gene locus. When fewer defective proteins are produced, the deleterious effect will be lower, which means lower dominance, noted *h*_1_ such that *h*_1_ < *h* (genotype (c) in Fig 1). Conversely, if more defective proteins are produced, the deleterious effect will be larger, which means a higher dominance, noted *h*_2_ such that *h*_2_ > *h* (genotype (b) in Fig 1). The values of *h*_1_ and *h*_2_ will depend on the strengths *e*_1_ and *e*_2_ of *E*_1_ and *E*_2_ alleles. In order to be more specific about this relationship, we note that phenotype-to-fitness relationship must verify two major properties: (1) the fitness must decrease as the proportion of deleterious proteins expressed increases and (2) the relationship between the fitness and the proportion of defective proteins expressed must be concave, as deleterious mutations are most often partially recessive [37], which means that fitness effects of deleterious mutations are lower than what would be expected in an additive (linear) situation. As a consequence, the relationship between fitness and the fraction of defective proteins expressed must be similar to the situation illustrated on Fig 2. A generic way to formulate these properties is to express fitness as a function of *f*_[*a*]_, the fraction of defective proteins, *h* and *s* with a concave monotonic power function:

$$W=1-sf_{\left[a\right]}^{-\frac{Log\left(h\right)}{Log\left(2\right)}},$$ (1)

which conveniently converges to the additive situation for *h* = 1/2. With such a relationship, and assuming that *e*_2_ > *e*_1_, we have:

$$\left\{ \begin{array}{l} h_{1}={\left(\frac{e_{1}}{e_{1}+e_{2}}\right)}^{-\frac{Log\left(h\right)}{Log\left(2\right)}}\\ h_{2}={\left(\frac{e_{2}}{e_{1}+e_{2}}\right)}^{-\frac{Log\left(h\right)}{Log\left(2\right)}} \end{array}\right.$$ (2)

Noting *Δh = (h*_*1*_ *+h*_*2*_ *)/2 –h*, one can use Jensen’s Inequality on the strictly concave fitness function to show that *Δh > 0*. The commonly accepted assumption that deleterious mutations are partially recessive (i.e. *h* is on average around 0.25 for mildly deleterious mutations, [37]) leads in our model to the outcome that polymorphism at the enhancer locus increases the mean dominance.

### Derivation of mutant enhancer frequency change over one generation

To study the evolution of enhancer strength, we are first looking for the frequency variation of the stronger *E*_2_ enhancer allele after one generation (noted as Δ*p*). To do so, a four-step life cycle is implemented, with diploid selection, meiosis, mutation and syngamy. The exact recursion is then linearized to leading orders, defining a parameter *ξ* << 1 and assuming (1) weak selection on the gene (*h s* is of order *ξ*), (2) very small mutation rate (*u* is of order *ξ* ^*2*^) and (3) small recombination rate *r* between enhancer and gene loci (of order *ξ*). Under those fairly generic assumptions, the frequency of the deleterious allele *a* (noted p_*a*_) is small (of order *ξ*) and the linkage disequilibrium (*D*_*EA*_) between the enhancer and the gene is small (of order *ξ*). *D*_*EA*_ is defined as positive when *E*_2_ *A* and *E*_1_ *a* haplotypes are over-represented (meaning that *D*_*EA*_ is positive when stronger enhancers are associated with beneficial alleles). We obtain, noting *p* the frequency of *E*_2_ and *q* the frequency of *E*_1_, the leading order of the frequency variation of *E*_2_:

$$\Delta p=-2\Delta hp_{a}pq\left(1-2p\right)s$$ (3a)

$$+D_{EA}\left[4hpq+\left(1-2p\right)\left(h_{2}q-h_{1}p\right)\right]\;s+o\left(\xi^{2}\right)$$ (3b)

The first term Eq (3a) corresponds to a direct selection on enhancers while the second term Eq (3b) is an indirect selection proportional to *D*_*EA*_.

The direct selection is negative when *E*_2_ is rare (since Δ_h_ > 0) and positive when it is frequent. Thus, it favors the most common allele at the enhancer locus. To understand this effect, it is useful to derive the mutation-selection equilibrium of *p*_*a*_, $p_{a}^{Eq}$. Using the same linearizing assumptions than for Δ*p*, assuming that the mutant stronger enhancer has just entered the population (thus neglecting *D*_*EA*_) and noting *δ*_*h*_ = *h*_2_−*h*_1_, one obtains:

$$p_{a}^{Eq}=\frac{u}{\bar{h}\;s}+o\left(\xi \right),$$ (4)

where $\bar{h}=h+2\;\Delta h\;p\;q$ is the average dominance in the population. Note that the polymorphism at the enhancer locus increases average dominance (since Δh > 0), which reduces the frequency of deleterious mutations. The direct selection term (3.a) actually stems from this effect on average dominance: fixation at the enhancer locus is similar to reducing average dominance, which is favorable. Like in models for the evolution of ploidy [38,39], direct selection favors the masking of deleterious mutations, but here this effect is frequency-dependent. We refer to this term as the ‘masking’ term.

The indirect selection is relatively straightforward since the expression within brackets in (3.b) can be shown to be always positive. That means that indirect selection has the same sign than *D*_*EA*_: it is positive when the stronger enhancer (*E*_2_) is preferentially associated with beneficial gene alleles (*D*_*EA*_ > 0 in this situation). The question turns now to determine the sign of *D*_*EA*_. To do so, we use a quasi-linkage equilibrium (QLE) approximation that requires that the forces causing frequency changes are weak relative to recombination rate [75]. This approximation usually breaks down at low recombination rates. However, because we are at mutation selection balance for the gene, and because the frequency change at the enhancer locus is small, an accurate approximation can be obtained by linearizing under the same assumptions as above, and keeping terms in both *rD*_*EA*_ and *sD*_*EA*_ [76]. We obtain $D_{EA}^{QLE}$:

$$D_{EA}^{QLE}=\frac{p\;q\;p_{a}\left[2\Delta_{h}\left(1-2p\right)+\delta_{h}\right]s}{2r+\left[h_{1}p^{2}+2pqh+h_{2}q^{2}\right]s}+o\left(\xi \right)$$ (5)

Note that this quasi-linkage equilibrium value does not diverge for small recombination rate. Noting that Δ_*h*_ is at most half as large as *δ*_*h*_, it is straightforward to show that $D_{EA}^{QLE}>0$, indicating that the indirect selection term is positive and favors stronger enhancers. This effect stems from the fact that *E*_*2*_ carrying chromosomes are more exposed to selection because of their increased average expression. They are thus purged more efficiently from deleterious mutations: as a consequence *E*_*2*_ alleles are most often found on, and hitchhike with, beneficial genetic background (they are associated to *A* alleles), which is beneficial for *E*_*2*_ alleles. We refer to this term as the ‘purging’ term.

The same model can be made with beneficial instead of deleterious mutations. In this case, we cannot assume that *p*_*a*_ << 1, since *a* alleles sweep to fixation. As a consequence the expression of Δ*p*, involves more terms depending on *p*_*a*_. However, Δ*p* can still be partitioned into a ‘masking’ and a ‘purging’ term like in Eq 3a and Eq 3b, respectively. Provided that beneficial mutations are dominant, and for any *p*_*a*_ value, the ‘masking’ term still favors the most frequent alleles, whereas the ‘purging’ term still favors stronger enhancers. Qualitatively, the model with beneficial dominant mutations and the model with deleterious recessive mutations give similar results concerning enhancer alleles’ frequency dynamics. Considering partially dominant deleterious mutations would lead to a moderately different outcome (the term 3.a will switch sign, causing more enhancer polymorphism), but this scenario is biologically much less relevant.

### Decrease of mean fitness

When a stronger enhancer spreads, it is favored by the purging term, but at the cost of unmasking deleterious mutations. Mean fitness equals *1–2u* when there is no variation at the enhancer locus (*p = 0* or *p = 1*). However, during the spread of an enhancer, mean fitness $\bar{W}$ decreases as can be readily seen from:

$$\frac{\partial \bar{W}}{\partial p}=-4\Delta h\left(D_{EA}+\left(1-2p\right)p_{a}\right)s,$$ (6)

which is negative around *p < ½* and positive around *p > ½*. This is due to the fact that deleterious mutations are unmasked when average dominance increases, before they have time to reach their lower mutation—selection equilibrium frequency. A temporary genetic burden appears: deleterious mutations are too frequent given their new mean dominance.

### Numerical calculation of mutant enhancer fixation probability

Because the selection coefficient on a new enhancer allele is frequency dependent (3.a), it is useful to obtain a more integrated measure of selection on enhancer alleles. One solution is to compute their probability of fixation *U* (which accounts for all frequency trajectories) and compare it to a neutral expectation. We computed it, for a mutant enhancer initially at frequency *p*_0_, using a diffusion approximation [77]:

$$U\left(p_{0}\right)=\int_{p=0}^{p_{0}}e^{-\int \frac{4N_{pop}\Delta p}{pq}dp}dp/\int_{p=0}^{1}e^{-\int \frac{4N_{pop}\Delta p}{pq}dp}dp$$ (7)

As the numerical integration calculated from Eq 7 relies on some assumptions, we use numerical simulations of mutant enhancer fixation or loss in a finite population to check the corresponding results. Fig 3 illustrates ratio of the probability of fixation of new enhancer alleles relative to 1/2*N*_*pop*_, the probability of fixation of a neutral allele. Numerical simulations were performed using a C++-program of an individual-based stochastic version of the model described above. There are *N*_*pop*_ individuals in the population. Each individual has two loci, enhancer and gene, with two alleles each. Alleles at the enhancer locus are encoded by a real value representing enhancer strength. Alleles at the gene locus carry either the wild-type allele or a deleterious allele of fitness effect *s*. The fitness of the diploid genotype is given by *w*_*1*_—*h*_*i*_ (*w*_*1*_—*w*_*2*_), were *h*_*i*_ is the dominance in this individual, which can vary depending on the genotype at the enhancer locus (following Eq 2) and where *w*_*1*_ is the fitness of the fittest of the two alleles (*w*_*2*_ the fitness of the other allele). At generation 0, all individuals are homozygote for the same enhancer allele and the wild-type gene allele. Then, 2000 generations of the life cycle (diploid selection, meiosis with recombination, mutation and syngamy) are performed. During these generations, there is no mutation on the enhancer locus. At each generation, the number of mutations on the gene is sampled in a Poisson Distribution with expectation 2*N*_*pop*_ *u* A corresponding number of alleles (sampled randomly in the population) is then assigned to be deleterious. We then generate the population of *N*_*pop*_ individuals at the next generation, accounting for selection, meiosis and random mating. For each individual in the next generation, we first determine its two parents. Two individuals of the current generation are sampled randomly. When chosen, each candidate is accepted with a probability equal to its fitness, or resampled. Once the two parents are identified, we sample one gamete in each of them (recombination occurring at a rate *r* between the two loci). After the 2000 generations, the deleterious allele frequency is close to the mutation-selection balance *u*/*hs*. A chromosome is then randomly chosen in the whole population and we assign it a new enhancer allele differing in strength. The new enhancer allele is then monitored until fixation or loss. Fixation probabilities were computed from 10000 runs of such simulations for each set of parameter values. The results presented on Fig 3 in the main text are obtained dividing those probabilities of fixation by that of a neutral enhancer in the same conditions (i.e. a mutant enhancer allele having the same strength than the resident allele).

**Fig 6:**
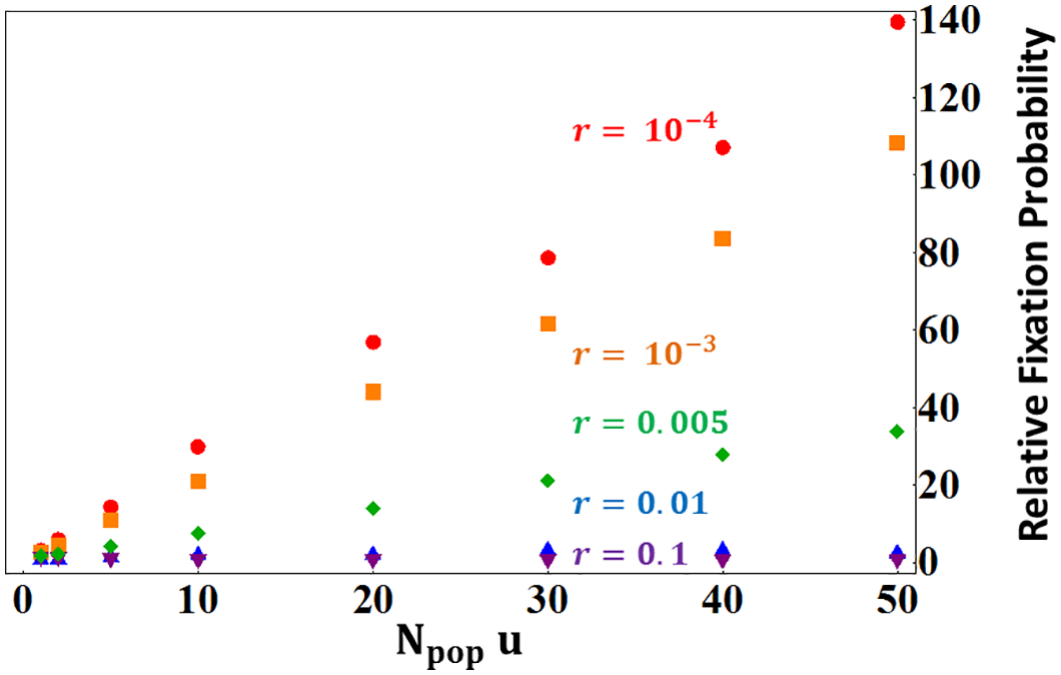
Fixation probabilities ratios of stronger enhancers (3:1 strength ratio compared to the resident allele) relative to that of neutral mutation (from Eq 7). Results were obtained for different recombination rates between the enhancer and the gene as indicated on the figure. Other parameter values are *s = 0*.*01*, *h* = 0.25.

Results showed on Fig 3 were obtained using the following parameters values: (1) dominance coefficient h = 0.25, (2) gene mutation rate *u* = 10^−3^, (3) population size *N*_*pop*_ = 10^3^, (4) selection coefficients *s* = 0.1 or 0.01 and (5) enhancer mutant strength three times larger or smaller than the resident allele. The *0*.*25* value of dominance is the most biologically plausible value for deleterious mutations [78]. Results illustrated are valid for other combination of *u* and *N*_*pop*_ provided *N*_*pop*_ *u* = 1. In other situations, the results would be magnified about 1 by a factor *N*_*pop*_ *u*. For instance in a very large population *N*_*pop*_ = 10^8^ with weaker gene mutation rate *u* = 10^−5^, we have *N*_*pop*_ *u* = 10^3^, so that tightly linked stronger enhancers would be ∼2.10^3^ more likely to fix than a neutral allele (instead of ∼2 more likely as illustrated on Fig 3 for *N*_*pop*_ *u* = 1). Fig 6 illustrates this behavior, where the fixation probability of stronger enhancers, relative to the neutral expectation, scales linearly with *N*_*pop*_ *u*.

### Numerical simulations of enhancer strength evolution

A simplifying assumption of the model described above is to consider a population with only two enhancer alleles. We also designed an infinite-allele version of the model to check the robustness of the conclusions and to study long-term enhancer strength evolution. The goal here is to study the evolution of the mean strength of a population of enhancers undergoing recurrent unbiased mutations. In order to be consistent, we also considered the gene locus as an infinite-allele locus undergoing recurrent deleterious mutations of various effects. The simulations performed on this model are designed as the fixation probability simulations with an individual-based stochastic model, except that we do not study fixation probabilities but long-term trait evolution. At each new generation, the number of mutations on the enhancer locus is drawn from a Poisson distribution with mean 2*N*_*pop*_ *u*_*E*_, *u*_*E*_ being the mutation rate on the enhancer locus per individual per generation. We denote *z*_*i*_ = Log(*e*_*i*_), the logarithm of enhancer strength of allele *i*. We consider that mutations alter the trait *z*_*i*_ such that after mutation it becomes *z*_*i*_ + *ε*, where *ε* is a normal deviate $\varepsilon ∼N\left(0,\sigma_{E}^{2}\right)$. We considered additive mutational effect on the logarithm of enhancer strength, in order to avoid that the mutational variance vanishes on mean enhancer strength when trait value increases. Indeed, in the model, only relative enhancer strength matters in the competition for expression. As a consequence, if mean enhancer strength increases in the long run, a constant mutational variance on the trait (not its Log) would tend to produce less and less differences between enhancers such that relative enhancer strength ratios will eventually tend to 1. Mutation as described above avoids this artifact and also ensures that enhancer strength always remains positive. To model mutations on the gene, the number of mutation events is drawn for each generation from a Poisson distribution with mean 2*N*_*pop*_ *u*_*A*_, *u*_*A*_ being the mutation rate on the gene locus per individual per generation. For each mutation event, the fitness effect of the new mutant allele is drawn from a negative exponential distribution with mean *s*.

In order to measure the rate of the ER process, we follow the population mean of *z* through time. This mean increases linearly with time and we use the slope of this linear increase to compute the mean doubling time (i.e. the time needed for mean enhancer strength to double). To obtain the mean doubling time, we first store at regular time points *z*_*1*_ *(i*,*j*,*t)* and *z*_*2*_ *(i*,*j*,*t)*, the logarithms of the strengths of both enhancers of individual *i* at generation *t* during iteration *j* (simulations are repeated 100 times). For each generation sampled, we calculate mean *z* over the whole population and over the different iterations:

$$\bar{z}\left(t\right)=\frac{1}{N_{it}}\sum_{j=1}^{N_{it}}\sum_{i=1}^{N_{pop}}\frac{z_{1}\left(i,j,t\right)+z_{2}\left(i,j,t\right)}{2N_{pop}}$$ (8)

As $\bar{z}$ increase linearly with time, we estimate its rate of increase using a linear regression. Doubling times *T* ^×2^ is computed as:

$$T^{\times 2}=\frac{Log\left(2\right)}{a}$$ (9)

where *a* is the slope of this regression. Results of this model are illustrated on Fig 4 (red curves) and were obtained using the following parameter values: (1) dominance coefficient *h* = 0.25, (2) mutation rates *u*_*A*_ = *u*_*E*_ = 10^−3^, (3) selection intensity s = 0.1, (4) recombination rate between the gene and its enhancer *r* = 10^−6^, (5) initial strength of enhancers *e*_0_ = 1, (6) population size *N*_*pop*_ = 5000, (7) number of iteration *N*_*it*_ = 100, (8) number of generations *N*_*gen*_ = 100000. Note that this model is a special case of model 2 and 3 presented below.

### Models including stabilizing selection on expression levels

#### Model 2: Stabilizing selection on relative expression levels among genes

Here we consider a model where, for example, the proteins are enzymes implied in bio-chemical chain reactions. In this case, the dosage relationship between enzymes plays a major role in the outcome of the reactions, and so potentially on the fitness of the individuals. In model 2, the absolute amount of proteins does not have any cost on fitness, but a departure from an optimum dosage between different proteins (produced from different loci) does. In reality, dosage relationships would probably involve several proteins, but we will consider only two for simplicity and because it probably corresponds to the maximum dosage constraint. With a larger sets of, say, *n* genes in dosage relationships, the increase in enhancer strength at a focal gene will alter its dosage relationships with *n*—1 genes. In principle, if each imbalanced gene pair contributes a cost then the overall cost should be larger for a larger set of genes. However, if, more plausibly, the set of co-regulated genes contribute to a specific biological function, then the fitness cost will be paid when this function is disrupted, but will not accumulate beyond this. Thus, it is robust to assume that the function will become more disrupted with a larger fraction of imbalanced gene pairs. Because this fraction equals 2/*n*, it is maximal for *n* = 2 (the number of imbalanced pairs is *n*-1 and the number of balanced pairs (*n*-1)(*n*-2)/2). Hence, as long as the fitness cost increases with the proportion of imbalanced genes pairs, the situation with only two genes is probably the most stringent. This model is designed similarly to model 1, but involves two major changes. First, we need two pairs of evolving enhancer/gene chromosomes to model the expression of the two types of proteins. We denote *z*_*ij*_ the log-strength of enhancer on chromosome *i* at locus *j*. We assume a recombination rate of *r* between the enhancers and the corresponding genes, and a recombination rate of 0.5 between the first gene and the second enhancer (which is equivalent to saying that there are two different chromosome pairs). Second, if we note *W* the fitness of an individual, $W_{A}^{j}$ the fitness of each diploid enhancer/gene set defined as previously and *W*_*E*_ a new fitness component taking into account the stabilizing selection on dosage relationship between the proteins, we have:

$$W=W_{A}^{1}\times W_{A}^{2}\times W_{E}$$ (10)

We denote *Z*_*j*_ the sum of the logarithmic strengths of the two alleles at the enhancer locus *j* (Z_*j*_ = z_1j_ + z_2j_). We define *W*_*E*_ as:

$$W_{E}=e^{-I{\left(Z_{1}-Z_{2}\right)}^{2}},$$ (11)

where *I* stands for the intensity of stabilizing selection on the dosage relationship. We apply stabilizing selection on *Z* to avoid any bias since mutations occur on *z*. Stabilizing selection favors an optimal phenotype where the two proteins are, for simplicity, equally produced. In order to biologically scale the intensity of stabilizing selection on expression (*I*) with the impact of deleterious mutations at the protein-coding loci, we introduce a scaling parameter *γ*. Considering that fitness reduction after one round of mutations on the gene is *u h s*, and that fitness reduction after one round of mutations on regulatory sequences is $1-e^{-I\times 4\sigma_{E}^{2}}$, we compute *I* such that:

$$1-\gamma \;u\;h\;s=e^{-I\times 4\sigma_{E}^{2}}$$ (12)

With this scaling, the cost of non-optimal expression of random mutations on enhancers is approximately *γ* time the cost of recurrent deleterious mutations on the gene. We run simulations for *γ* values of *0*, *1*, *5* and *10*. Fig 4 illustrates these simulations with the same parameter values than for model 1.

#### Model 3: Stabilizing selection on absolute expression level on a focal gene

Here, we consider the second case where, contrary to model 1, enhancer polymorphism causes variation in expression levels. In this case, stabilizing selection acts directly on absolute expression levels. Any increase or decrease from the optimal amount of proteins is deleterious. In this situation, we also allow for trans-acting regulatory factors (referred to as transcription factors below, TFs) to evolve as well, as they may compensate for the evolution of stronger enhancers. In model 3, the absolute level of protein expression depends on the strengths of enhancers and TFs. The goal here is to investigate if enhancer strength evolution is limited by the constraint on absolute expression levels or if an open ended coevolution can take place between enhancers, whose strength tends to increase due to the ER process, and the TFs, for which we predict that they should coevolve such as to maintain optimal protein expression. For this model we designed simulations with three loci: the TF locus, the enhancer locus and the gene locus. TF locus is not localized on the same chromosome, i.e. it recombines freely with the two other loci, *r*_*TE*_ = 0.5. It influences expression on the gene in *trans*, i.e. each allele at this locus interacts with the two homologous enhancer alleles. Following the same notation as previously, we note Z_1_ and Z_2_ the logarithmic strengths of enhancer and TF loci, respectively. Without loss of generality, we assume that optimal amount of proteins is produced when Z_1_ + Z_2_ = 0 (meaning that the overall strength of TFs exactly opposes the overall strength of enhancers):

$$\{\begin{array}{c} W=W_{A}\times W_{E}\\ W_{E}=e^{-I{\left(Z_{1}+Z_{2}\right)}^{2}} \end{array},$$ (13)

The intensity of the stabilizing selection *I* is scaled as in the previous model. Like for the enhancer locus, the number of mutations on the TF locus is drawn from a Poisson distribution with mean 2*N*_*pop*_ *u*_*T*_; and these mutations additively change the Log of the trait. Results on Fig 4 are presented in terms of doubling time as previously and were obtained with the same parameter values than before.

### Effect of inbreeding on the ER process (model 4)

In model 4, we want to study modifications in escalation rates resulting from variation in the mating system. Here, we use the same assumptions than in model 1 except that each individual gets a probability *p*_*s*_ to self-fertilize. Self-fertilization is modelled by sampling the second gamete from the same diploid parent than the first. Doubling times are calculated as previously and are reported on Fig 5.

## Supporting Information

S1 Fig Ratio of fixation probabilities of mutations altering enhancer strength relative to that of a neutral mutation (y-axis) in relation with the logarithm of the recombination rate between the enhancer and the gene (x-axis). In blue, the mutant enhancer (*e*_*2*_) is ten times stronger than the wild-type enhancer (*e*_*1*_). In red, it is three times stronger. In green, it is 1.5 times stronger. This figure was obtained using the analytical version of model 1 (see methods), with following parameters: partial recessivity *h = 0*.*25*, selection intensity *s = 0*.*1*, *Npop u = 1*. Results show that, at short recombination distances, selection for stronger enhancers is larger for larger enhancer strength differences. However, at larger recombination distances, selection for stronger enhancers decreases faster for larger strength differences. Consequently, the recombination rate limit after which stronger enhancers are selected against is larger for smaller enhancer strength differences.
(TIF)
