# Supplementary figures and images for: Enhancer Runaway and the Evolution of Diploid Gene Expression
Source: PLoS Genet. 2015 Nov 12;11(11):e1005665. doi: 10.1371/journal.pgen.1005665 (PMC4642963; doi:10.1371/journal.pgen.1005665)

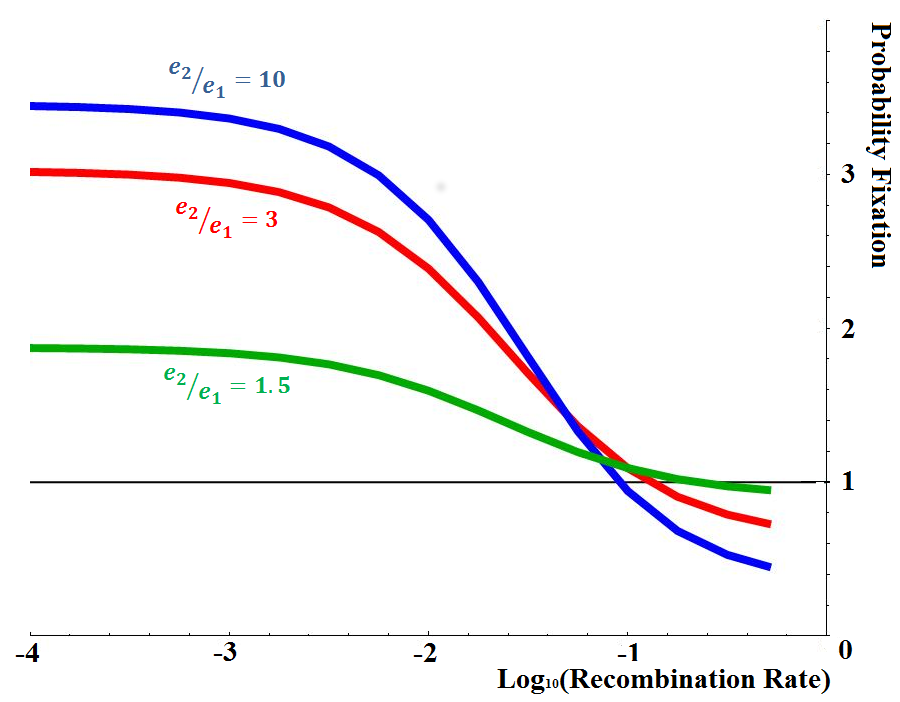

Supplement: S1 Fig — In blue, the mutant enhancer (e 2) is ten times stronger than the wild-type enhancer (e 1). In red, it is three times stronger. In green, it is 1.5 times stronger. This figure was obtained using the analytical version of model 1 (see methods), with following parameters: partial recessivity h = 0.25, selection intensity s = 0.1, Npop u = 1. Results show that, at short recombination distances, selection for stronger enhancers is larger for larger enhancer strength differences. However, at larger recombination distances, selection for stronger enhancers decreases faster for larger strength differences. Consequently, the recombination rate limit after which stronger enhancers are selected against is larger for smaller enhancer strength differences. (TIF) [file pgen.1005665.s001.tif]
